# Hemispherical differences in the shape and topography of asteroid (101955) Bennu

**DOI:** 10.1126/sciadv.abd3649

**Published:** 2020-10-08

**Authors:** M. G. Daly, O. S. Barnouin, J. A. Seabrook, J. Roberts, C. Dickinson, K. J. Walsh, E. R. Jawin, E. E. Palmer, R. Gaskell, J. Weirich, T. Haltigin, D. Gaudreau, C. Brunet, G. Cunningham, P. Michel, Y. Zhang, R.-L. Ballouz, G. Neumann, M. E. Perry, L. Philpott, M. M. Al Asad, C. L. Johnson, C. D. Adam, J. M. Leonard, J. L. Geeraert, K. Getzandanner, M. C. Nolan, R. T. Daly, E. B. Bierhaus, E. Mazarico, B. Rozitis, A. J. Ryan, D. N. Dellaguistina, B. Rizk, H. C. M. Susorney, H. L. Enos, D. S. Lauretta

**Affiliations:** 1 The Centre for Research in Earth and Space Science, York University, Toronto, ON, Canada.; 2 Johns Hopkins University Applied Physics Laboratory, Laurel, MD, USA.; 3 MDA Ltd., Toronto, ON, Canada.; 4 Southwest Research Institute, Boulder, CO, USA.; 5 Smithsonian Institution National Museum of Natural History, Washington, DC, USA.; 6 Planetary Science Institute, Tucson, AZ, USA.; 7 Canadian Space Agency, St. Hubert, QC, Canada.; 8 Teledyne Optech Inc., Toronto, ON, Canada.; 9 Université Côte d’Azur, Observatoire de la Côte d’Azur, CNRS, Laboratoire Lagrange, Nice, France.; 10 Lunar and Planetary Laboratory, University of Arizona, Tucson, AZ, USA.; 11 NASA Goddard Space Flight Center, Greenbelt, MD, USA.; 12 University of British Columbia, Vancouver, BC, Canada.; 13 KinetX Inc., Simi Valley, CA, USA.; 14 Lockheed Martin Space, Littleton, CO, USA.; 15 School of Physical Sciences, Open University, Milton Keynes, UK.

## Abstract

We investigate the shape of near-Earth asteroid (101955) Bennu by constructing a high-resolution (20 cm) global digital terrain model from laser altimeter data. By modeling the northern and southern hemispheres separately, we find that longitudinal ridges previously identified in the north extend into the south but are obscured there by surface material. In the south, more numerous large boulders effectively retain surface materials and imply a higher average strength at depth to support them. The north has fewer large boulders and more evidence of boulder dynamics (toppling and downslope movement) and surface flow. These factors result in Bennu’s southern hemisphere being rounder and smoother, whereas its northern hemisphere has higher slopes and a less regular shape. We infer an originally asymmetric distribution of large boulders followed by a partial disruption, leading to wedge formation in Bennu’s history.

## Teaser

A lidar model of asteroid (101955) Bennu has a north-south asymmetry in shape and evidence of an early partial disruption.

## INTRODUCTION

NASA’s Origins, Spectral Interpretation, Resource Identification, and Security–Regolith Explorer (OSIRIS-REx) mission will return a sample from asteroid (101955) Bennu (*1*). To provide data for selecting the sampling site, as well as geological context for the sample itself, the spacecraft carries a diverse suite of instruments. These include a camera suite (*2*), optical spectrometers (*3*, *4*), an x-ray spectrometer (*5*), and a scanning laser rangefinder (or lidar) (*6*); we focus here on data collected by the lattermost instrument, the OSIRIS-REx Laser Altimeter (OLA), which was provided to the mission by the Canadian Space Agency. OLA is the first scanning lidar instrument to fly on a planetary mission. OLA’s task was to collect altimetric data for global geophysical and local topographic analyses, as well as studies of other mission data in the context of the asteroid’s shape. In addition to supporting the sample acquisition objective of the mission, these efforts provide insight into the origin and evolution of Bennu.

The shape of Bennu has been described using an Earth observation–based radar model (*7*) and a stereophotoclinometric (SPC) model based on early mission image data (*8*). The radar model was created with a facet resolution of 20 m. Its mean radius and volume are 246 ± 10 m and 0.062 ± 0.006 km^3^, respectively. The radar data indicated a rotation period of 4.297 ± 0.002 hours and a pole axis at (87°, −65°) ± 4° in J2000 equatorial coordinates. The SPC model updated these estimates using a model facet resolution of 0.8 m. The SPC model had a mean radius of 244 ± 0.09 m and volume of 0.0615 ± 0.0001 km^3^, in agreement with the radar model. The rotation period was 4.296057 ± 0.000002 hours, with a period rate of change of −1.02 ± 0.15 s per century (*8*). A center-of-mass/center-of-figure offset was identified of (1.38 ± 0.04, −0.43 ± 0.07, and −0.12 ± 0.27) m in *x*, *y*, and *z* (*8*).

Earlier investigations (*8*–*11*) determined that Bennu is a rubble-pile asteroid with a top-like shape It bulges (i.e., has its largest radius) at the equator due to the centrifugal force acting on the rubble pile, but this feature is more muted than on Ryugu (*12*), a similar type of carbonaceous asteroid to Bennu, and appears square-shaped with rounded corners when viewed from a pole. Four longitudinal (north-south) ridges were identified in the northern hemisphere, with at least two extending to the south pole (*8*). By “ridges,” here, we mean elongated and subtly delineated mound-like features that are not necessarily pronounced in the topography but that are evident in shape analyses (*8*). Evidence was found for grooves indicative of surface fracturing, as well as mass wasting indicative of downslope flow (*8*–*11*). The longitudinal ridges, grooves, and surface displacement indicate that despite being a rubble pile, Bennu exhibits some internal cohesiveness or stiffness (*8*).

Here, we explore Bennu’s shape by using a 20-cm-resolution OLA-based global digital terrain model (GDTM). Unlike the SPC approach, OLA provides a direct measurement of shape and topography that is not inferred from images. As OLA is an active measuring device, its observations are not dependent on the presence of illumination, solar incidence angles, or other observation constraints that impact the quality of the SPC-based models, particularly at high latitudes (±50°). The OLA-derived GDTM more accurately represents the topography of the surface features of Bennu, capturing intermediate-sized (<2 m) boulders that were smoothed in the SPC model, as seen in side-by-side comparisons with images in the methods section of (*8*). The higher fidelity of the OLA GDTM relative to previous shape models of Bennu provides the detail necessary to understand the origin of observed hemispherical differences, especially at high latitudes, with their implications for Bennu’s global structure and surface evolution.

## RESULTS

### Modeling Bennu with altimetric data

Over a 5-week period in July and August 2019, the OSIRIS-REx spacecraft globally mapped the surface of Bennu from a near-terminator quasi-circular orbit with a radius of <1 km [Orbital B (*1*)], relative to the asteroid center of mass. OLA’s scanning capability and high measurement rate (10 kHz) made it possible to achieve global high-density coverage of Bennu during this period, in orbital geometries with low orbital velocities of tens of centimeters per second and ranges near 700 m. OLA collected measurements of Bennu in 5.5-min continuous blocks (scans), each of which overlapped the previous scan in a sequence that typically consisted of 15 to 20 scans (e.g., figs. S1 and S2). Each scan was approximately 182 × 175 mrad (azimuth × elevation) in extent with a nominal 100-μrad spot spacing, although the scans extended in the direction of the spacecraft ground-track (elevation direction) owing to the spacecraft velocity over the scan duration. Each scan consists of more than 3.3 million measurements that are well referenced with respect to the other measurements in the same scan after correction for the relative asteroid and spacecraft motions. The end result is ≈ 2.7 × 10^9^ measurements with average spot spacings below 5 cm globally, other than in areas where shadowing prevented measurement. These individual scans were assembled into the GDTM using the methods described in (*13*, *14*) and in Materials and Methods.

The global point cloud acquired by OLA was meshed into a global surface using two methods. For global analysis, the point cloud was preprocessed using Generic Mapping Tools (GMT) (*15*) to obtain a single median surface estimate for each latitude and longitude on Bennu. This provides a relatively uniform sampling of the surface for global analyses based in geodetic coordinates and excellent understanding of misregistrations between scans. Details of this approach are presented in (*14*). The negative consequence of this method is poor modeling of boulder overhangs, as the GMT method assumes all OLA observations occur at zero emission with the observer looking straight down at the surface, whereas the OLA scans frequently view the sides and overhangs of boulders. For analyses where the fidelity of individual features is required in either local or global form, the global point cloud was meshed into a surface using the Poisson reconstruction meshing technique (*16*) that supplements the techniques described in (*14*) and preserves overhangs where supporting data exist.

**Fig. 1:**
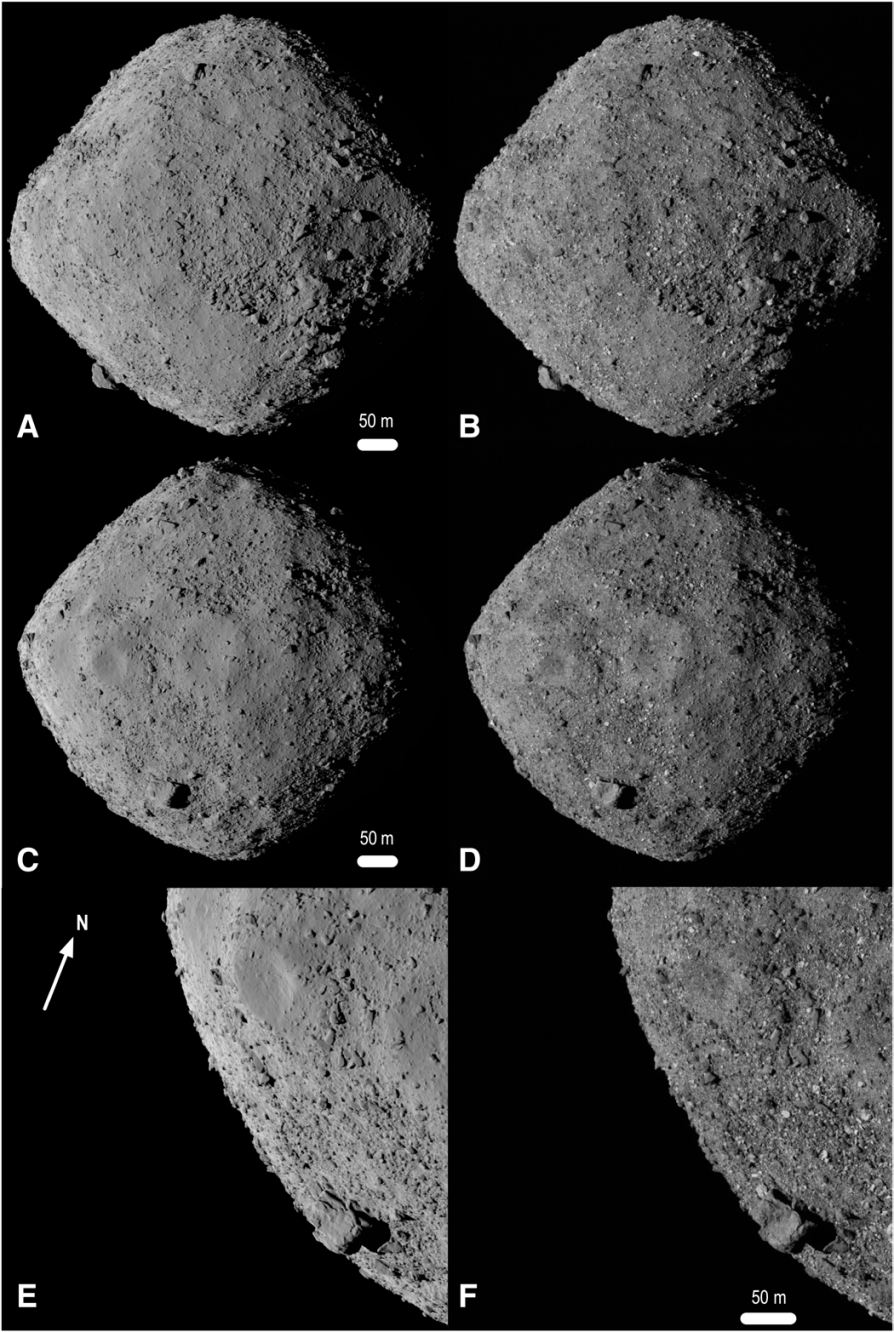
The 20-cm-resolution GDTM of Bennu. (**A**, **C**, and **E**) Views of the rendered OLA model and (**B**, **D**, and **F**) corresponding images acquired by OSIRIS-REx Camera Suite (OCAMS) (*2*). Images in (B) and (D) were taken by the MapCam imager on 13 December 2018 at 1:01:48 UTC (subobserver location −5, 156°E) and 1:59:00 UTC (subobserver location −7, 232°E), respectively. The image in (F) was acquired by the PolyCam imager on 2 December 2018 at 8:18:29 UTC (off-limb subobserver location). Aside from albedo differences (bright boulders in the OCAMS images), the OLA model and the images are nearly indistinguishable. The model is Poisson-reconstructed from the point cloud and rendered into an image. The pole axis is vertical and Bennu north (+*z*) is up in (A) to (D). The model can be viewed over Bennu’s full rotation in movie S1.

The resulting GDTM has facet vertex separations of ≈20 cm (Fig. 1) and compares well with images of Bennu. Assessments that include detailed optical navigation efforts, adoption of absolute OLA ranges, and updated estimates of the asteroid GM indicate that the accuracy of the OLA GDTM is better than ±20 cm. The precision of the GDTM, based on differences between overlapping OLA scans, is on the order of ±1.25 cm and very close to the precision measured by OLA on the ground (*6*). The OLA GDTM has an average radius that is 41 ± 20 cm smaller than that of the SPC model (*17*) described in (*8*) and provides a geometric baseline for assessing the physical size of Bennu and the related geopotential quantities that depend on it. These parameters, along with updates to the pole and rotation-state parameters, are in Table 1. To investigate the characteristics of Bennu evident in the model, including apparent differences between the northern and southern hemispheres, we examine the large-scale structures on Bennu, surface regularity and roughness, localized topographic features, and other, nontopographic information that can inform our interpretation.

**Table 1:** Physical parameters of Bennu. Updated physical parameters of Bennu as determined from the OLA GDTM, using the 0.878-m GMT-derived version of the model for consistency with previously reported results (*8*, *9*, *38*). RA, right ascension; DEC, declination.

**Parameter**	**Previous GDTM value**	**OLA GDTM value**
GDTM number of facets	3,145,728	3,145,728 m
GDTM average facet length	0.878 m	0.878 m
Average radius*	244 ± 0.09 m	242.22 ± 0.15 m
Best-fit ellipsoid†	(252.78 ± 0.05) × (246.20 ± 0.09) × (228.69 ± 0.12) m	(252.37 ± 0.09) x (245.91 ± 0.09) x (228.37 ± 0.09) m
Volume	0.0615 ± 0.0001 km^3^	0.061354 ± 0.00006 km^3^
Surface area	0.7820 ± 0.004 km^2^	0.78740 ± 0.0004 km^3^
Bulk density	1,190 ± 13 kg m^–3^	1194 ± 3 kg m^–3^
Pole	RA = (85.65 ± 0.12)°; dec = (−60.17 ± .09)°	RA = (85.45218 ± 0.00034)° , DEC = (−60.36780 ± 0.00010)°
Period (equatorial J2000)	4.296057 ± 0.000002 hours	4.2960015 ± 0.0000018 hours
Period rate of change	−1.02 ± 0.15 s per century	−1.02 ± 0.15 s per century
Center-of-mass/center-of-figure offset	(1.38 ± 0.04, −0.43 ± 0.07, −0.12 ± 0.27) m	(1.31 ± 0.03, 0.46 ± 0.04, 0.22 ± 0.01) m
Gravitational acceleration	−0.000058 to 0.000080 m s^–2^ Weighted mean = 0.0000595 ± 0.0000001 m s^–2^	−0.0000748 to 0.0000806 m s^–2^ Weighted mean = 0.0000542 ± 0.0000001 m s^–2^
Facet slopes‡	0.0° to 92.0°, weighted mean = 17° Median = 16.3°	0.0° to 113.0°, weighted mean =33° Median = 24.6°

*,†Previous uncertainties were assessed based primarily on model variations, whereas the present assessment uses all available information including spacecraft navigation data.

‡Differences driven by improved resolution of boulders. Weighted mean without boulder contribution is 22° with a median of 18.6°.

### Shape

The OLA GDTM of Bennu exhibits differences in shape between the northern and southern hemispheres, which is not unexpected given that shape asymmetry can be produced during asteroid reaccumulation processes following the disruption of a parent body or shape readjustments following spin-up as a result of a thermally driven process called the Yarkovsky-O’Keefe-Radzievskii-Paddack (YORP) effect (*18*). Some of the hemispherical differences are observable in Approach-phase full-disk images and the image-based GDTM of Bennu (*8*) but were not fully recognized or assessed, owing to uncertainties above about ±50° latitude related to limited quantities of images suitable for SPC.

**Fig. 2:**
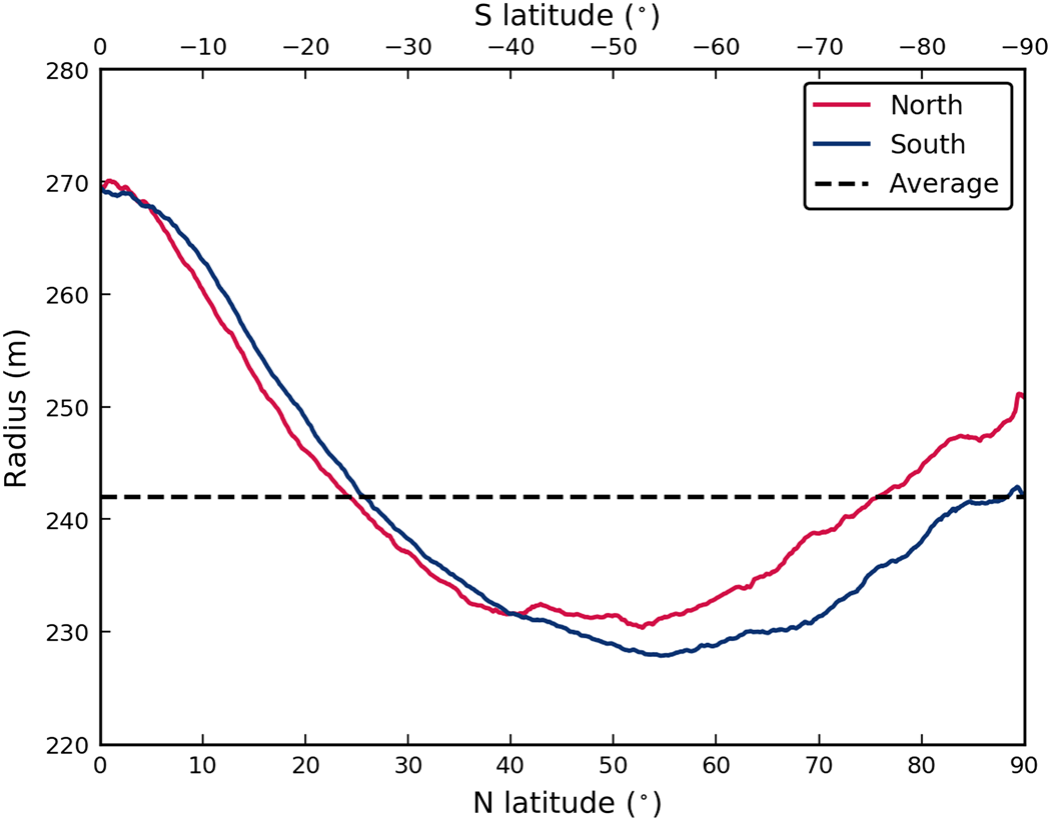
The median radius of Bennu. Differences in shape between the northern and southern hemispheres are evident, with the northern hemisphere exhibiting higher curvature than the south.

The median radii of Bennu averaged for all longitudes (Fig. 2) show that the equatorial bulge has a radius 27.2 ± 10.3 m larger than the mean radius. In the midlatitudes, the radius decreases relative to the mean radius by 14.6 ± 3.4 m at −54.9° and 11.9 ± 6.0 m at 52.8°. The radius in the north reaches the mean radius at 76.1° and then exceeds it by 8.59 ± 0.35 m at the pole. The northern hemisphere is narrower with greater curvature (smaller local radius) relative to the southern hemisphere, which is closer to a sphere, with a radius at the pole that essentially matches the mean (difference of 0.11 ± 0.35 m).

**Fig. 3:**
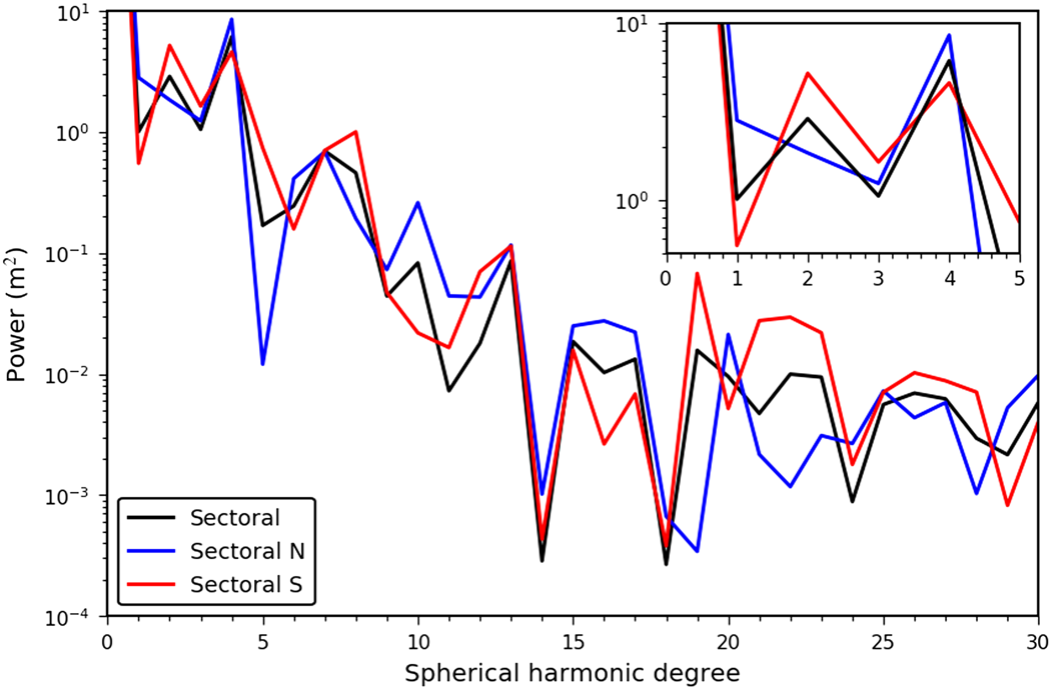
A sectoral spherical harmonic analysis. The hemispheres show similar power in the degree-4 term that indicates the longitudinal ridges, but the southern hemisphere has a higher degree-2 term that represents material obscuring the ridges in the south.

We performed a global spherical harmonic analysis of Bennu’s shape [Fig. 3 (sectoral) and fig. S6 (zonal)]. Spherical harmonics are a set of basis functions that are suitable for harmonic analysis of objects and fields of roughly spherical symmetry. The harmonic content can be considered with respect to sectoral components (east/west nodes), zonal (north/south nodes), and tesseral components (more complex nodes).

Sectoral degree-4 terms that indicate fourfold longitudinal symmetry in Bennu’s structure were identified in the image-based global shape analysis (*8*). This symmetry is due to the four north-south ridges evident in the northern hemisphere and at the equator.

The image-based global analysis (*8*) suggested that two of these four ridges extend through the equator to high southern latitudes. In the present analysis, we split the OLA GDTM at the equator, mirrored the hemispheres, and analyzed each hemisphere’s harmonic components separately (*19*). This allowed us to see that a degree-4 term is evident in both mirrored hemispheric models with similarly high power (Fig. 3). Our analysis indicates that all four ridges are present in the south with a similar topographic contribution that was not evident in the previous analyses (*8*). However, portions of the ridges are obscured in the south by surface material that contributes to the higher degree-2 harmonic, indicative of twofold longitudinal symmetry. This finding has implications for Bennu’s internal structure, considering the global extent of these features and the underlying strength required to support them.

### Topography

**Fig. 4:**
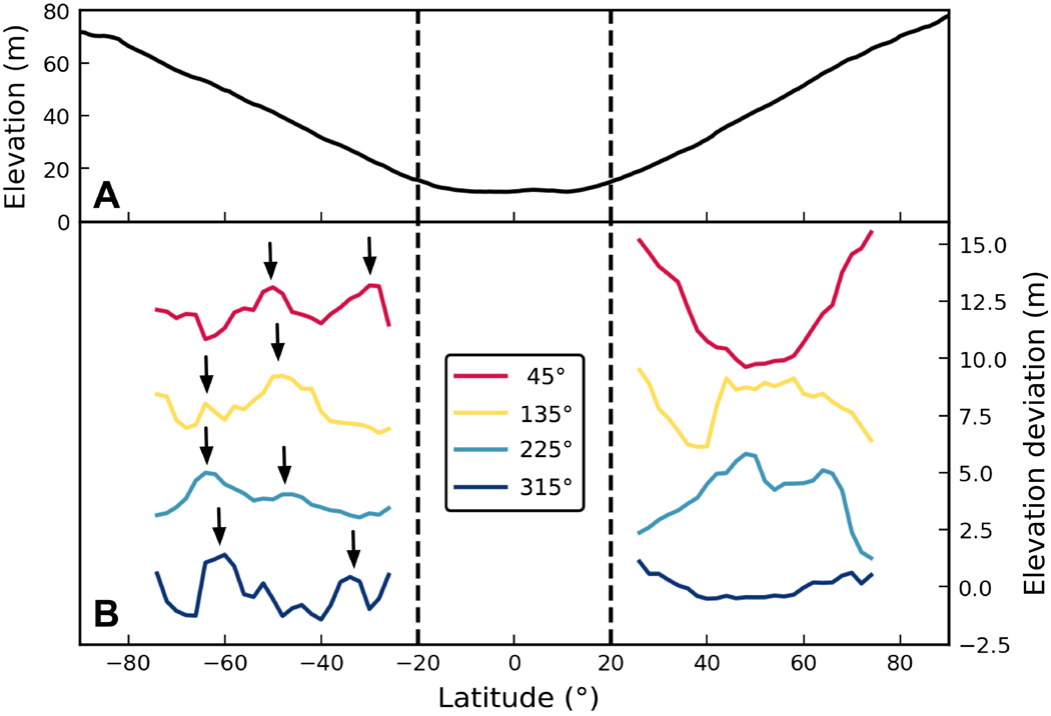
The geopotential elevation of Bennu. (**A**) Global results median-averaged over all longitudes show that the equatorial region between +20° and −20° has a flat profile. (**B**) The linearly detrended elevation as a function of latitude over four 90° longitude sectors (centered where indicated in the legend and each vertically offset in +4-m elevation increments for clarity) indicates greater regularity in the south, with terrace-like features (indicated by arrows) extending over multiple adjacent sectors and exhibiting similar latitudinal spacing.

Asteroid topography is captured by surface elevation, which includes the effects of the asteroid’s gravity coupled with its spin state. It defines the downslope direction (*8*) and its analysis provides insights into recent surface processes. We investigate two of Bennu’s topographic regions located above latitudes of ±20° to quantify the nature of the north-south dichotomy (Fig. 4A). These regions are steeper relative to the equatorial region that is contained between +20° and −20° and thus are more susceptible to surface displacements that may influence the resulting shape of Bennu. The equatorial region is the sink for downslope movement of material and is, therefore, essentially flat. Furthermore, the equatorial region is governed by unique dynamical processes in that it falls within the rotational Roche lobe, where material is energetically bound to the surface (*10*) and does not reflect the hemispherical dichotomy.

Differences between the two hemispheres can be seen when assessing the elevation above the equatorial region. With changes of under 1 m/degree, the elevation profiles flatten noticeably relative to the north within 15° of the pole. The north pole is 5 m, or 5.9% of the full dynamic range of the elevation, higher than the south. Although changes in elevation from the equator to the poles appear to follow a linear trend, subtle topographic deviations are evident. These are enhanced when the elevation profiles are linearly detrended (Fig. 4B). Splitting these detrended data into longitudinal sectors of 90°, the sectors of the north show little correlation to one another, with elevation deviations that extend ±3 m from the trend line. Conversely, the southern sectors have smaller changes of ±1 m from the trend line, with features that extend across multiple sectors. In the southern sectors, there are elevation features that represent regular surface failures with similar relief and latitudinal spacing. These terrace-like forms have been posited to be the result of slow creep modifying the surface as the asteroid’s rotation rate increases (*20*). The northern hemisphere has terraces, but they do not exhibit similar regularity with latitude and are shorter in longitudinal extent.

### Surface roughness

**Fig. 5:**
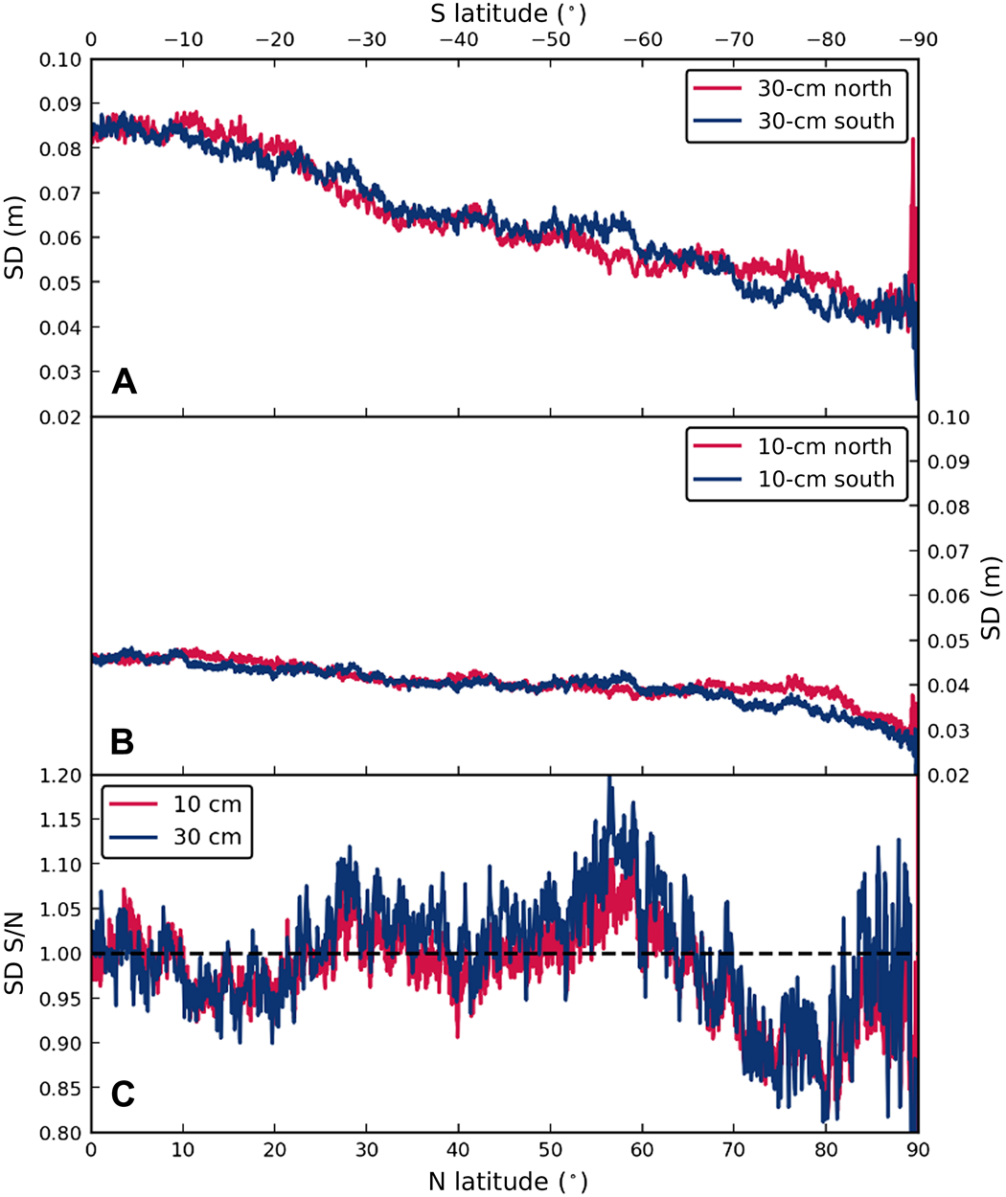
Binned SD of the GDTM as a measure of roughness. The SD between OLA returns is binned into (**A**) 30-cm and (**B**) 10-cm bins and medianed over all longitudes as a measure of surface roughness at these two scales. (**C**) The ratio of south and north SDs for 10-cm and 30-cm bins. The rapid changes in the roughness at poles are in part due to the smaller number of bins and in part to the presence of boulders.

Surface roughness can be used to infer differences in the geological processes that have acted on a surface. This approach has been used to analyze several planetary bodies [e.g., (*21*–*25*)]. As a measure of surface roughness, we divided the complete OLA point cloud—which was decimated to make the 20-cm GDTM, and therefore coregistered with it—into surface patches and assessed the standard deviation (SD) between the OLA returns within each patch. The patches (or bins) were set at 10 cm by 10 cm or 30 cm by 30 cm to capture roughness properties at different horizontal scales (Fig. 5). These horizontal scales of roughness show more roughness variations across Bennu than seen at longer scales and delineate albedo and textural differences visible in OSIRIS-REx Camera Suite [OCAMS (*2*)] images.

Both hemispheres show a spatial trend in roughness at the two horizontal scales considered, moving from high latitudes, with relatively low roughness, to the equator, the highest roughness (Fig. 5). At the 10-cm scale, the northern hemisphere roughness is 0.4 cm greater (or 9.6% of the full dynamic range of the SDs) from near the pole to near 65° when compared to the southern counterpart region. At the 30-cm scale, this pattern is also present, with higher roughness in the northern hemisphere of 1 cm (or 11.4% of the full dynamic range of the SDs). This pattern reverses briefly between 55° and 60° N and S, where the south is rougher by about 10% of the full dynamic range regardless of horizontal extent. Within this latitude range, the greater roughness in the south is probably driven by the presence of boulders and terraces.

A hemispheric difference is evident in the distribution of boulders greater than or equal to 30 m in long dimension. Table S1 identifies the locations and size of the 17 boulders on Bennu that meet this criterion, all of which are in the low-reflectance subset of Bennu’s boulder population (*26*). All but two are in the southern hemisphere.

**Fig. 6:**
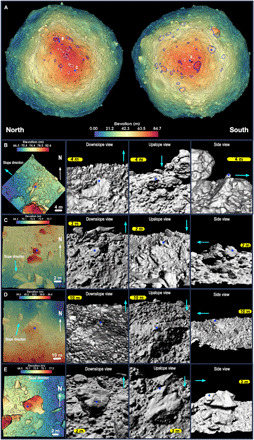
Boulder locations and relationships with surrounding material. (**A**) Hemispherical views of the elevation of Bennu overplotted on the OLA GDTM. Boulders that are visibly retaining material on the upslope side are outlined in blue, whereas perched boulders are outlined in white. Local DTMs in (**B**) and (**C**) show examples of northern boulders that are dynamically emplaced, specifically toppled (B), and imbricated (piled on one another) (C). Local DTMs in (**D**) and (**E**) show examples of southern boulders that are stationary or may have moved with the surrounding surface: (D) a material-retaining boulder and (E) a perched boulder. Locations of the local DTMs on the GDTM are shown by the letters. Cyan arrows show the downslope direction. The blue star indicates the same location on each boulder view.

Figure 6 (left) shows our mapping of individual boulders in both hemispheres. To more closely examine these features, we created local DTMs from high-density OLA point clouds (Fig. 6, right, and movies S2 to S5) of 96 boulders. Some boulders are entrained and/or embedded in the regolith, whereas others are perched on the surface. The boulders (≥30 m) support and retain upslope material (e.g., Fig. 6C). The perched boulders do not appear to have been substantially disturbed, and there is no evidence of material accumulating against them (e.g., Fig. 6D). If these small boulders have moved, they were displaced with the surrounding finer material.

The local DTMs of boulders are of sufficient quality in three dimensions that sedimentological indicators of rock angularity and roundedness (*27*) can be applied. We do not find any preferential occurrence of such morphological attributes for the perched boulders—indicating that no favored origin of deposition for these features can be assigned. This is similarly true for the entrained and/or embedded boulders. These boulders are samples of the general population. However, large material-retaining boulders are rounded.

In the south, most of the boulders <10 m close to the pole appear to be perched, whereas larger- and lower-latitude boulders are supporting upslope material [see (*28*) for a corroborating image-based analysis] and have overhangs on the downhill side, where it is likely that finer material has preferentially moved downslope. These material-retaining boulders are often located near regional slope breaks that delineate parts of the southern hemisphere. The most prominent are the ≥30-m boulders that help define the transition between the southern and equatorial regions.

In the north, as in the south, perched boulders are present, but they are less frequent and do not extend as low in latitude as those in the south. The northern perched boulders also reach larger diameters (up to 22 m). Large material-retaining boulders are also observed in the north but, again, are less common. As in the southern hemisphere, material-retaining boulders frequently delineate the breaks in slope in the northern hemisphere, but the slope breaks tend to be located closer to the pole in the north than in the south. Because large boulders are less common in the north, the resulting breaks in slope are not as uniform as those in the south, as seen in the detrended elevation (Fig. 4).

The boulders in the north show more evidence of dynamics (toppling and downslope movement) than in the south. Several of the local DTMs of these northern boulders show features indicative of entrainment in past surface flows. For example, we observe (Fig. 6, A and B) boulders toppled onto neighboring ones, as well as imbrication, where clusters of boulders are piled on each other in the upslope direction. Also present in the north are a small number of the boulders that appear buried on their downslope side. These cases occur on the outer sides of crater rims and could be ejecta that dug their front ends into the regolith as they were deposited from ballistic flight. The upslope sides of these rocks are not buried. These types of boulder geologies were not identified in the south.

**Fig. 7:**
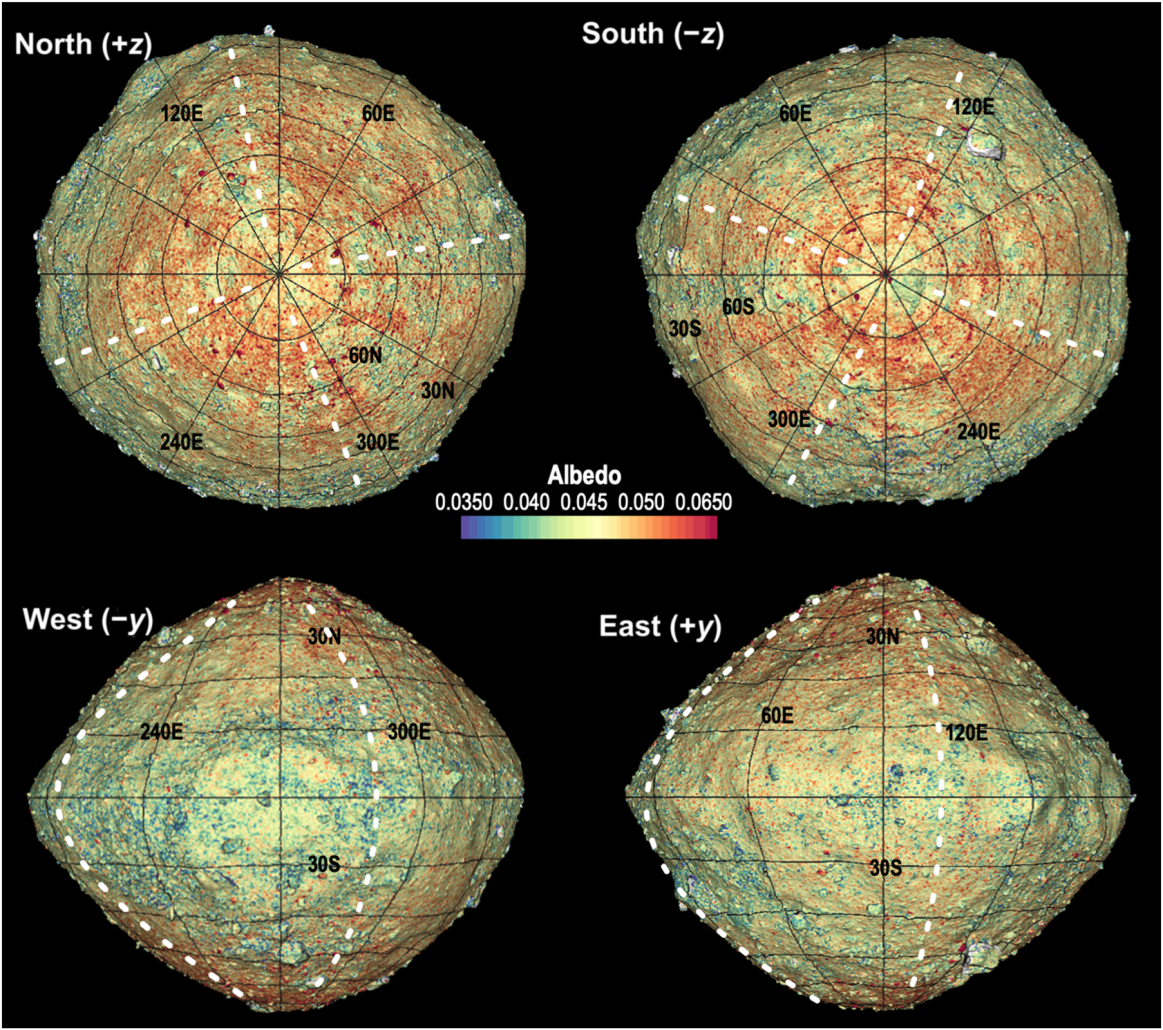
OLA-derived albedo registered to a shape model slightly shaded to indicate topography. Dark (green and blue) areas are more prevalent in the southern hemisphere, with dark “spokes” present between the longitudinal ridges (approximate locations shown in white). The sides of some rocks do not have meaningful OLA lidar albedo data owing to observation angle limitations and so appear gray in some views.

Rockslides and falls are present in the north, usually between longitudinal ridges (*8*). Two examples can be seen in the northern hemispherical view of Fig. 7 near 0°E and 310°E, respectively, where dark and rough rocky regions with respective median slopes of 39° and 38° are apparent. Such deposits are usually located between the north-south ridges. In the south, they are much less common. One similar rocky deposit is visible near 350°E; others are less distinct (e.g., at 135°E and 195°E) with smaller downslope extent.

### Other evidence of hemispherical differences

OLA acquires a peak-height measurement of the returned pulse that can be mapped over Bennu’s surface (*6*, *29*). Because this measurement returns the peak pulse height and not the integrated energy, it is not strictly a measurement of albedo at the OLA wavelength of 1064 nm, considering that surface roughness over the size of a ≈ 7-cm laser spot can stretch the pulse and lower the peak height for the same energy. A similar effect can occur for large incidence angles, although the nominally nadir observation geometry in this dataset should largely confine such an effect to the sides of boulders. For our purposes here, we refer to the peak height measurements as albedo, recognizing that they may also be indicative of other effects.

This albedo dataset, superimposed on the shape model (Fig. 7), shows that Bennu has large regions with predominantly higher or lower surface albedo, consistent with image analysis (*26*). The surface generally falls into the 2 to 6% range in our dataset with an average of 4.5%. We also observe a small number of very high-albedo boulders identified in image data and posited to be exogenic by (*30*).

The spatial patterns of low-albedo areas differ between the two hemispheres. In the southern hemisphere, we observe large-scale, low-albedo–delineated surface features, some of which are correlated with latitudinal high-slope areas and boulders. These surface features generally occur in the transition region between the sloped southern cap and the flatter equatorial region. In the northern hemisphere, fewer such localized large low-albedo features exist, although some large dark areas with scattered fine-scale material identified early as rock slides or falls are present, mainly between the north-south ridges.

The large low-albedo features in the southern hemisphere extend in a north-south direction toward the south pole, appearing as a spoke-like pattern in a polar stereographic projection (Fig. 7, top right). In fig. S7, the approximate longitudinal extents of the north-south ridges consistent with the OLA model and as identified in (*8*) are reported along with the approximate longitudinal extent of the low-albedo spokes. Only one of these radial patterns is associated with the longitudes of the north-south ridges; the other four are associated with the regions between the ridges. The one that is correlated with a ridge contains a large outcrop-like feature that may be representative of the underlying structural material. All but one of the southern boulders contribute to the low-albedo spokes.

In addition to the hemispheric differences in the albedo as measured by OLA, Bennu’s thermal inertia varies as a function of latitude: The equatorial region has the highest values, and the poles have the lowest (*31*). In the north, a relatively constant positive thermal inertia slope extends from the polar low of 280 J m^−2^ K^−1^ s^−1/2^ to the equatorial high of almost 330 J m^−2^ K^−1^ s^−1/2^. In the south, the polar thermal inertia is a lower 265 J m^−2^ K^−1^ s^−1/2^ and remains near that value until −45° latitude and below the north-polar high until −30° latitude (*31*).

## DISCUSSION

The distinctions we identified between the northern and southern hemispheres of Bennu suggest fundamental differences in surface properties and subsurface structure. The southern hemisphere is rounder and smoother at surface roughness scales of 10 and 30 cm. Furthermore, its variations in elevation are smaller (±1 m versus ±3 m) and longitudinally more regular than those in the north, and its near-constant thermal inertia values (*31*) suggest greater homogeneity in surface material.

Four longitudinal ridges extend pole to pole, but in the southern hemisphere, two of these are obscured by surface material. The southern hemisphere exhibits a spoke-like pattern of large, low-albedo (as derived from OLA data) areas, which tend to occur between the longitudinal ridges. The lower-elevation interridge areas may be collecting fragments of the large, dark boulders common in the south as they break down over time.

The longitudinal ridges on Bennu have been suggested to be structural and indicative of cohesion and strength (*8*). Their subdued nature, especially in the south, suggests that they are old relative to the resurfacing processes that may have led to the hemispherical differences we have identified. The ridges are expressed in the equatorial region and are largely responsible for the rounded-square equatorial shape. The presence of some of the largest craters on Bennu near the equator suggests that the equatorial bulge is old and, therefore, that the ridges are of similar age.

The longitudinal ridges may be indicative of a “wedging” rotational failure in Bennu’s history, similar to those modeled for rubble piles in (*32*). In those simulations, the amount of cohesion controls the number of cohesive elements (wedges), and ridges occur at or near the centers of these elements. In some simulations of near-spherical asteroids, the cohesive elements are roughly equal-sized sectors with pole-to-pole extents. We thus suggest that Bennu underwent a spin-related wedging event, in which material collapsed toward the asteroid center, exposing the largest structural units at the surface. No disturbed or deformed craters are observed on Bennu that can be traced to this process, further implying that the longitudinal ridges are at least as old as the surface age inferred from the crater population (*11*).

The evidence that the wedging occurred early in Bennu’s history indicates it must have arisen during a past spin-up that led to partial disruption of the asteroid. The formation of the wedges would have led to inertial or topographic changes that impeded further disruption by changes in the moments of inertia of the asteroid and/or alterations to the YORP drivers of shape and surface thermo-optical properties. The wedging event may have happened during the reaccumulation process that formed Bennu following its parent body’s disruption (*33*) or in any subsequent YORP-driven spin-up event (*18*).

The southern hemisphere’s four-sector elevation profiles exhibit regularly spaced terrace-like features extending over at least two sectors. These terrace-like forms are further indicated by median calculations for sectoral analyses (Fig. 4). We assert that more regular and evenly spaced slips are compatible with a more homogeneous regolith that exhibits similar strength over the majority of the southern cap. This would allow for surface material slip to occur with some regularity as a function of elevation as Bennu undergoes its current spin-up. In the north, the paucity of large material-impeding boulders has precluded the development of such homogeneity; more material may have moved downslope to better expose the longitudinal ridges.

The large material-impeding boulders in the southern hemisphere are present primarily between longitudinal ridges. Therefore, they may have been present before and during the longitudinal ridge–creation process. Their rounded, often lumpy appearance provides further evidence for their old age. These boulders may be of sufficient size that they become embedded in surrounding material that inhibits movement, or they may indicate underlying structural strength in the southern hemisphere near-surface that does not exist in the north.

The finding that thermal inertia is almost constant over the southern cap—whereas, in the northern hemisphere, the values increase linearly toward the equator (*31*)—suggests that a mechanism is active in the north that alters or sorts the regolith with downslope movement. [It might also be active at the equator, but mechanisms proposed by (*10*, *34*) could be the driver at low latitudes.] This mechanism could be related to size, and/or composition, and/or porosity. For a size-related mechanism, considerable changes to the size-frequency distribution of regolith within the few-centimeters-thick thermal skin depth (*31*) would be necessary to be evidenced in the thermal inertia.

Bennu’s low and high values of thermal inertia correspond respectively to low and high albedos, as measured by image analysis (*26*, *31*). A mechanical sorting process may be at work where density and mechanical strength preferentially retain more high-albedo and high-thermal-inertia material near the surface in substantial downslope movements, of which we see more evidence in the northern hemisphere. This would result in more low-albedo material remaining in the south, consistent with our observation.

The southern hemisphere is smoother particularly at the 10-cm scale for latitudes >67°, which is more relevant than the 30-cm scale to the thermal inertia, given the skin depths of a few centimeters. The higher roughness in the north is suggestive of more dynamic processes and, in particular, the sorting process driven by mass movement, where larger size fractions (in this case, >10 cm) provide additional surface roughness, up to the 30-cm scale, at which the roughness between the two hemispheres becomes more similar. In many mass movements, larger objects rise to the surface via the Brazil nut effect (*35*) and sort by increasing size in the flow direction. Such increasing size sorting is visible on Bennu at some of the rock deposits seen at the base of ridges in the northern hemisphere and is further reflected in the increasing surface roughness estimates with decreasing latitude (Fig. 5). Alternatively, the roughness differences may be dominated by preferential loss of weak, low-strength, low-albedo material with downslope movement, as suggested by the thermal inertia results discussed above. The smoother southern surface is consistent with the retaining of fine material that is able to settle into a shape closer to that of an equipotential surface (Fig. 2).

The boulder population suggests an explanation for the observed homogeneity in the south. Boulders that appear perched or nonembedded are more prevalent in the south and extend to lower latitudes than those in the north. We interpret this as further evidence of less downslope flow and more settling of rocks in concert with the supporting fines in the south, as opposed to processes that move material toward the equatorial sink in the north. Large boulders in the southern hemisphere are holding back material and allowing some of the smaller boulders closer to the pole to remain perched and relatively undisturbed. We posit that the retention of fines and the decreased significance of downslope processes that sort and differentiate the surface material are the reason for the more homogenous southern hemisphere.

The hemispherical asymmetry in the surface distribution of large boulders, and its consequence for the surface processes that resulted in the different shapes of the hemispheres, is probably not an outcome of wedging. More likely, the conditions leading to this asymmetry were set up during the early reaccumulation following Bennu’s parent body disruption. Numerical simulations of reaccumulation after disruption [e.g., (*32*)] show that asymmetry may occur, even when an asteroid that appears to have a spinning top shape is formed after catastrophic disruption. We propose that subsequent downslope movement has uncovered pre-existing boulders in the areas between ridges, which, in the south, act to impede further downslope movement of material to the equator and subdue the expression of the ridges at the surface. In the north, the paucity of large retaining boulders has led to a more dynamic deposition environment with little retention and more uncovering of the old longitudinal ridges.

The shape and topography of Bennu suggest a formation in which a north-south asymmetry was established in the population of large boulders. During this event, or thereafter, Bennu’s equatorial bulge was likely formed by spin-related processes. Coincident with or closely following bulge creation, Bennu’s rotation was accelerated either through reaccumulation processes or by the YORP effect, resulting in partial disruption into four wedges that formed the structural elements of the north-south ridges. The underlying structural strength after the wedging event was dominated by axial strength supported by the ridges and supplemented by the population of large blocks and possible subsurface population of similar material. Subsequent surface refiguring by downslope material movement was hindered in the south by these large, material-retaining boulders and possible underlying structure, resulting in a more homogeneous southern hemisphere cap. In the north, the paucity of large boulders allowed for greater downslope flow and more material- or size-sorting processes. It also uncovered the ridges that are obscured by surface material in the south.

## MATERIALS AND METHODS

### Global DTM

The GDTM of Bennu was created using a series of individual 5.5-min continuous OLA scans taken at a measurement rate of 10 kHz between 1 July and 5 August 2019. Each of these scans (e.g., fig. S1) overlapped the previous scan in a sequence that typically consisted of 15 to 20 scans (e.g., fig. S2). OLA scans are referenced with unique scan identifiers (scan IDs), and this dataset consists of scan IDs 4000 through 4910, with scans 4711 through 4729 excluded due to an instrument power-up anomaly and subsequent operational execution using obsolete software.

The scans were corrected for spacecraft position, spacecraft pointing, and Bennu’s rotation using the SPICE framework (*36*, *37*). They were then assembled into a self-consistent global point cloud by iteratively minimizing the differences between matched features in overlapping scans as described in (*13*, *14*) until the scan mismatches were balanced over all the scans. The method depends on each scan being well constructed to minimize long-wavelength shape errors. The quality of the resulting model point cloud was initially evaluated by gridding the data using GMT (*15*) into ^1^/_32_-degree bins and plotting the SD in each bin (fig. S3). Poor registration of individual scans would be apparent in this as clearly delineated, anomalously high SDs with boundaries that correspond to individual scans. Visible scan edge artifacts are not evident in fig. S3.

The deviations in fig. S3 are instead dominated by surface features at these scales, with the largest deviations representing the edges of boulders. To achieve these low SDs in the GDTM, an additional correction to the scan data was required. Initial models created using this process resulted in 1 to 1.5 m of compression at the equator and a similar expansion at the poles. Investigations into the origin of this long-wavelength shape error were conducted after initial analyses that compared approximately orthogonal scans near the poles suggested a scanning mirror scale error of compression in the azimuthal scan axis and/or expansion in the elevation axis. This error was verified, and empirical corrections were derived by evaluating the goodness of fit in the development of global models. Figure S4 shows the result of this analysis and its sensitivity. The resulting correction factors that were applied to the scan angles to develop this model were 1.0073 for the azimuth and 1.0000 for the elevation.

An additional check on the quality of the model is provided by the shifts required to assemble it from the J2000 frame positions derived from spacecraft positions. Tight constraints on spacecraft position are possible, in this case, as the spacecraft was orbiting Bennu in the near-terminator orbit and many orbits can be used to understand the local dynamics of the spacecraft. For this model, the average bias was a negligible ≈2 cm with no latitudinal bias (fig. S5), providing additional support for the data quality and scale of the resulting point cloud.

The global point cloud was meshed into a surface using the Poisson reconstruction meshing technique (*16*) that supplements the techniques described in (*14*) to preserve overhangs where supporting data exist.

## Supplementary Material

abd3649_Movie_S2.mp4

abd3649_Movie_S3.mp4

abd3649_Movie_S4.mp4

abd3649_Movie_S1.mp4

abd3649_Movie_S5.mp4

abd3649_SM.pdf

## SUPPLEMENTARY MATERIALS

Supplementary material for this article is available at http://advances.sciencemag.org/cgi/content/full/6/41/eabd3649/DC1
