# Supplementary material for: Hemispherical differences in the shape and topography of asteroid (101955) Bennu
Source: Sci Adv. 2020 Oct 8;6(41):eabd3649. doi: 10.1126/sciadv.abd3649 (PMC7544500; doi:10.1126/sciadv.abd3649)
Supplement: abd3649_SM.pdf [file abd3649_SM.pdf]

Science Advances
AAAS

advances.sciencemag.org/cgi/content/full/6/41/eabd3649/DC1

Supplementary Materials for

Hemispherical differences in the shape and topography of asteroid (101955) Bennu

M. G. Daly*, O. S. Barnouin, J. A. Seabrook, J. Roberts, C. Dickinson, K. J. Walsh, E. R. Jawin, E. E. Palmer, R. Gaskell, J. Weirich, T. Haltigin, D. Gaudreau, C. Brunet, G. Cunningham, P. Michel, Y. Zhang, R.-L. Ballouz, G. Neumann, M. E. Perry, L. Philpott, M. M. Al Asad, C. L. Johnson, C. D. Adam, J. M. Leonard, J. L. Geeraert, K. Getzandanner, M. C. Nolan, R. T. Daly, E. B. Bierhaus, E. Mazarico, B. Rozitis, A. J. Ryan, D. N. Dellaguistina, B. Rizk, H. C. M. Susorney, H. L. Enos, D. S. Lauretta

*Corresponding author. Email: dalym@yorku.ca

Published 8 October 2020, *Sci. Adv.* **6**, eabd3649 (2020)
DOI: 10.1126/sciadv.abd3649

The PDF file includes:

Figs. S1 to S7
Table S1
Legends for movies S1 to S5

Other Supplementary Material for this manuscript includes the following:

(available at advances.sciencemag.org/cgi/content/full/6/41/eabd3649/DC1)

Movies S1 to S5

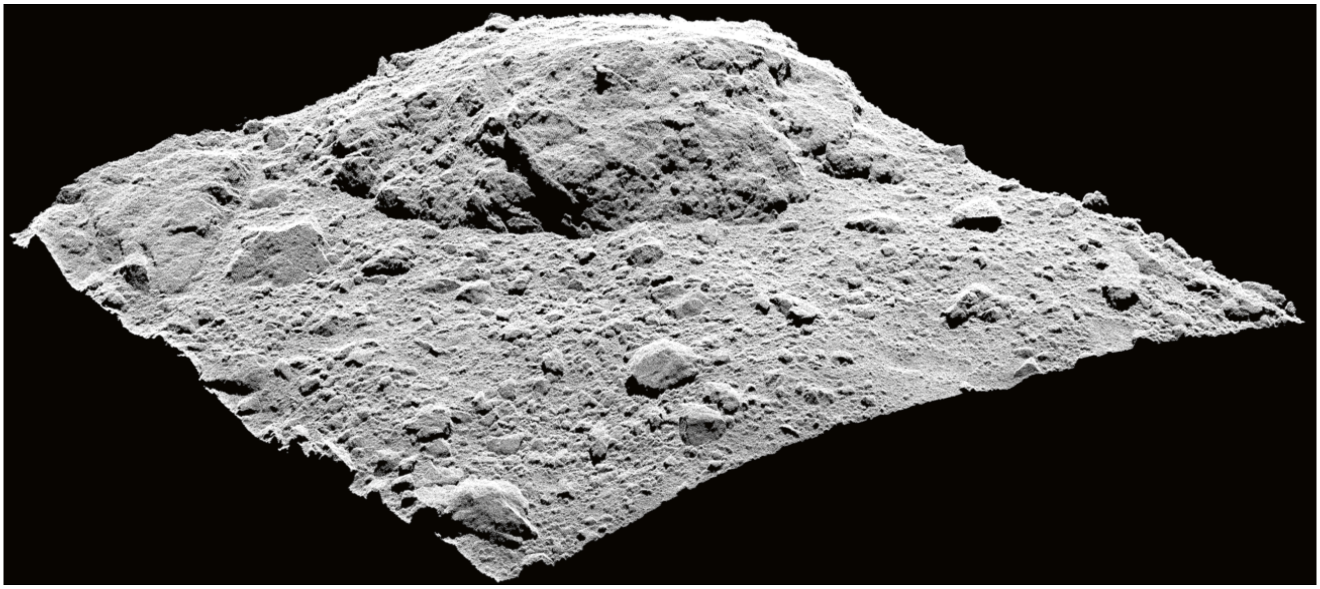

Fig. S1. A representative single 5.5-minute scan of the surface of Bennu. Scans are taken at a range of ≈700 m from the surface with average spot spacings of ≈7 cm. They each consist of more than 3.3 × 10^6 points. The scan shown here (ID 4067) is centered at 28.4° longitude and –17.5° latitude with corner coordinates from the bottom corner and proceeding clockwise of: (39.4, –-5.1), (49.5, –33.6), (13.9, –30.6) and (12.9, –4.6). Each scan's dimensions vary based on altitude, but typically the projected dimensions are 125 m × 125 m.

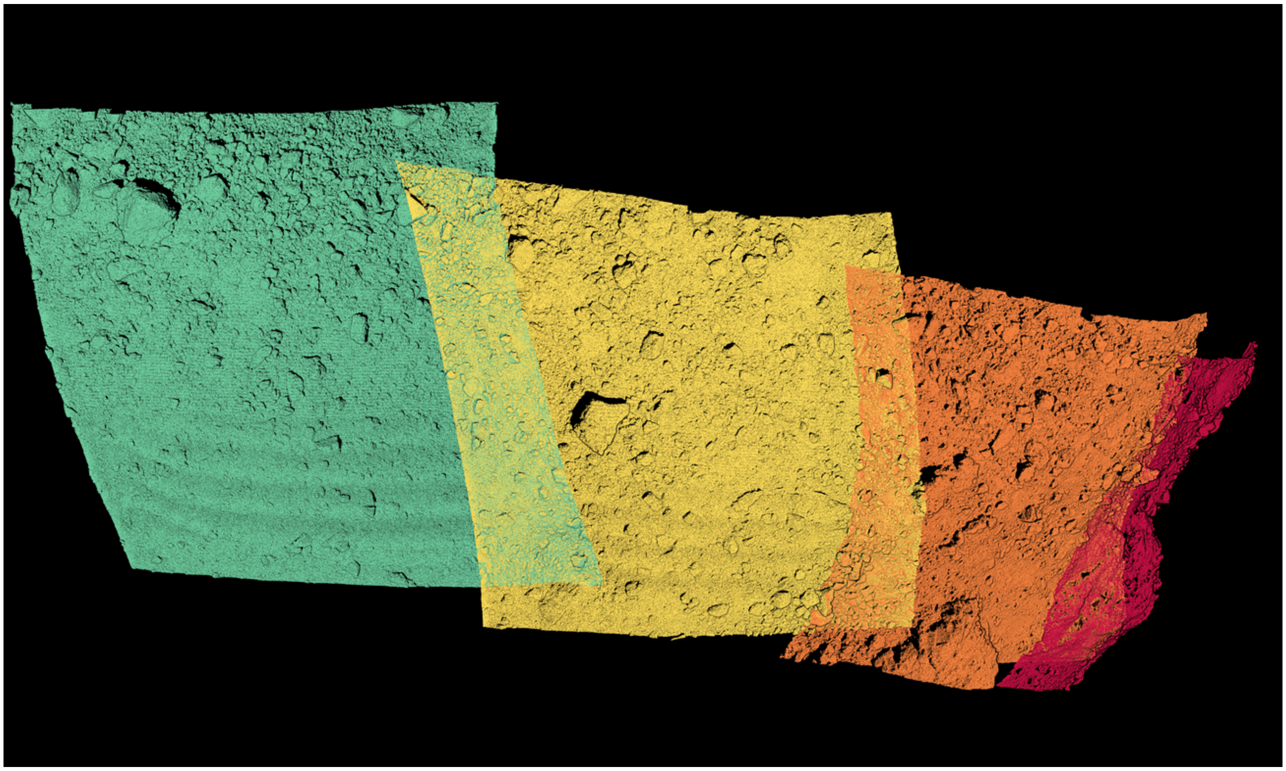

Fig. S2. A series of overlapping 5.5-minute scans being co-registered. Approximately 900 such scans were stitched together to create the GDTM, using the method described in (*13*). The scans were sequentially collected and are centred at (longitude, latitude), from left to right, of (316.5°, –3.6°), (340.4°, –8.3°), (4.2°, –13.1°), (28.0°, –17.5°), with corresponding IDs, from left to right, of 4070, 4069, 4068, and 4067.

.

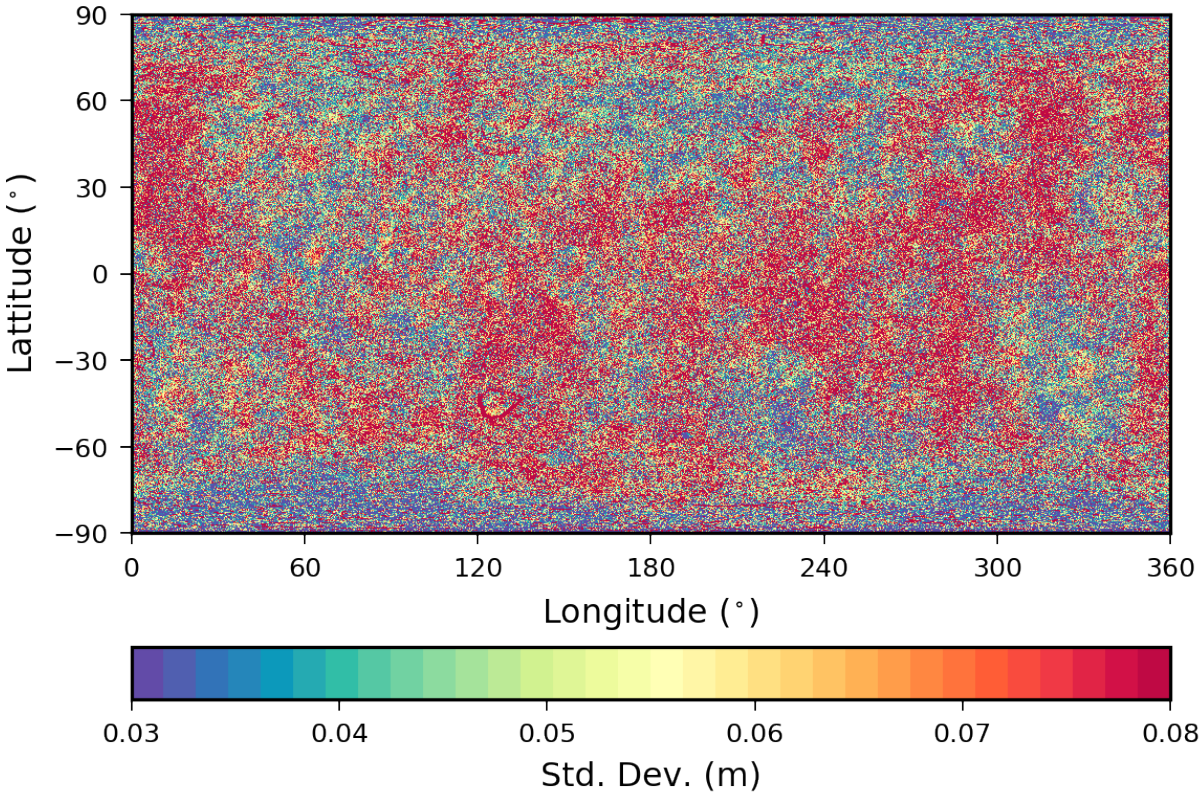

Fig. S3. The processed global OLA point-cloud data gridded into 1/32-degree bins. The standard deviations in each bin are controlled by surface features and not by data registration, as no scan edges are visible.

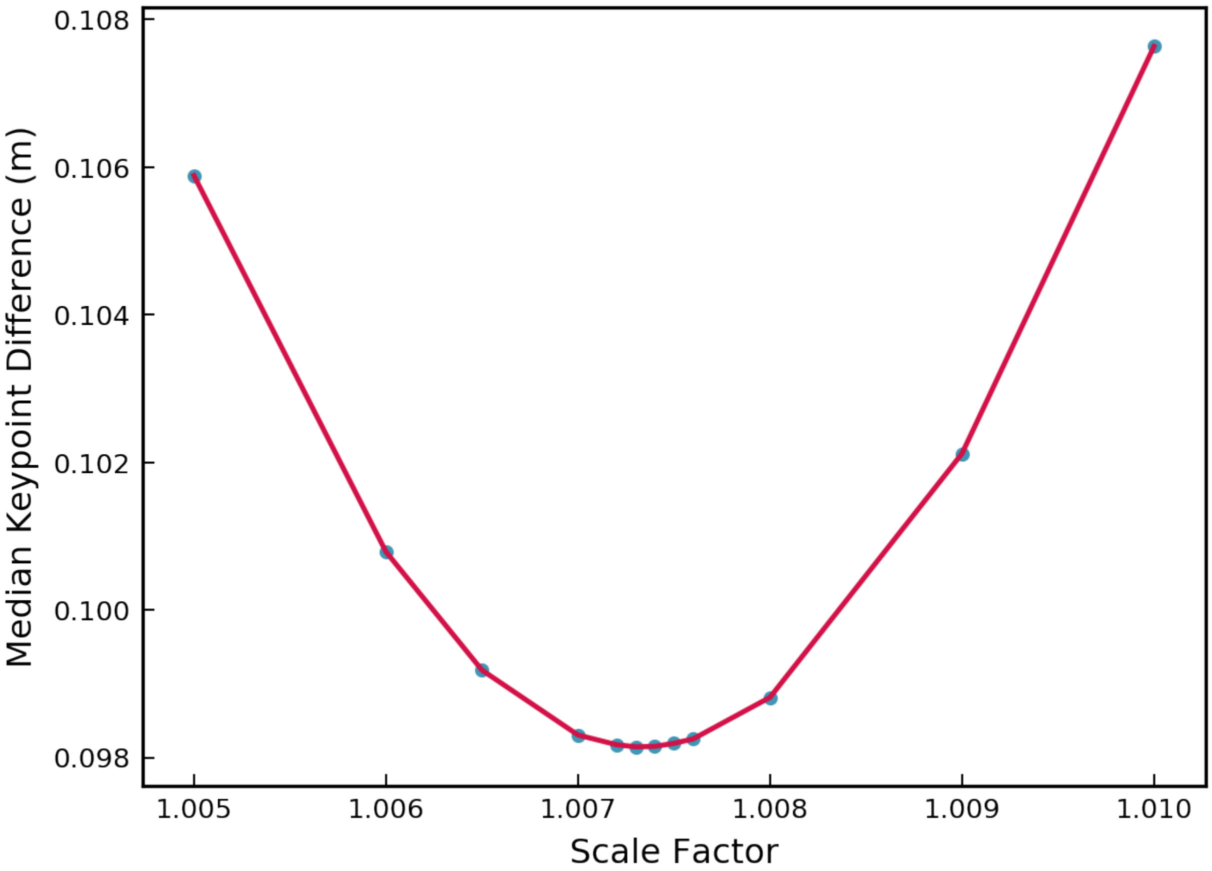

Fig. S4. OLA mirror scale factor analysis. The scale factor in the azimuthal mirror direction required to maximize consistency with the other scans is plotted against global mean distance between matched scan features. A scaling of 1.0073 is indicated.

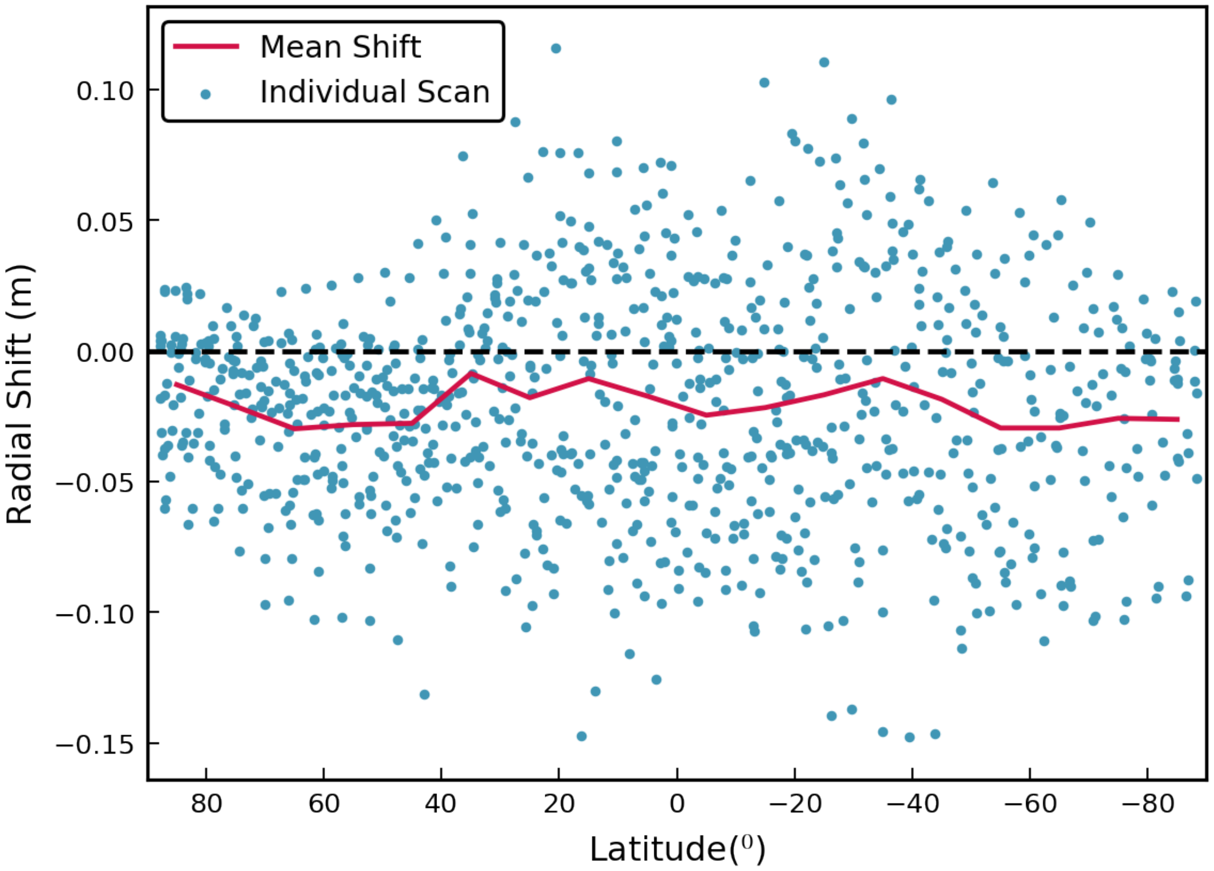

Fig. S5. The radial difference between scans located by spacecraft position and those determined by co-registration. The radial shift of individual scans shows no latitudinal bias and has a mean near zero, which implies that the model is consistent with orbital constraints developed by the spacecraft navigation team.

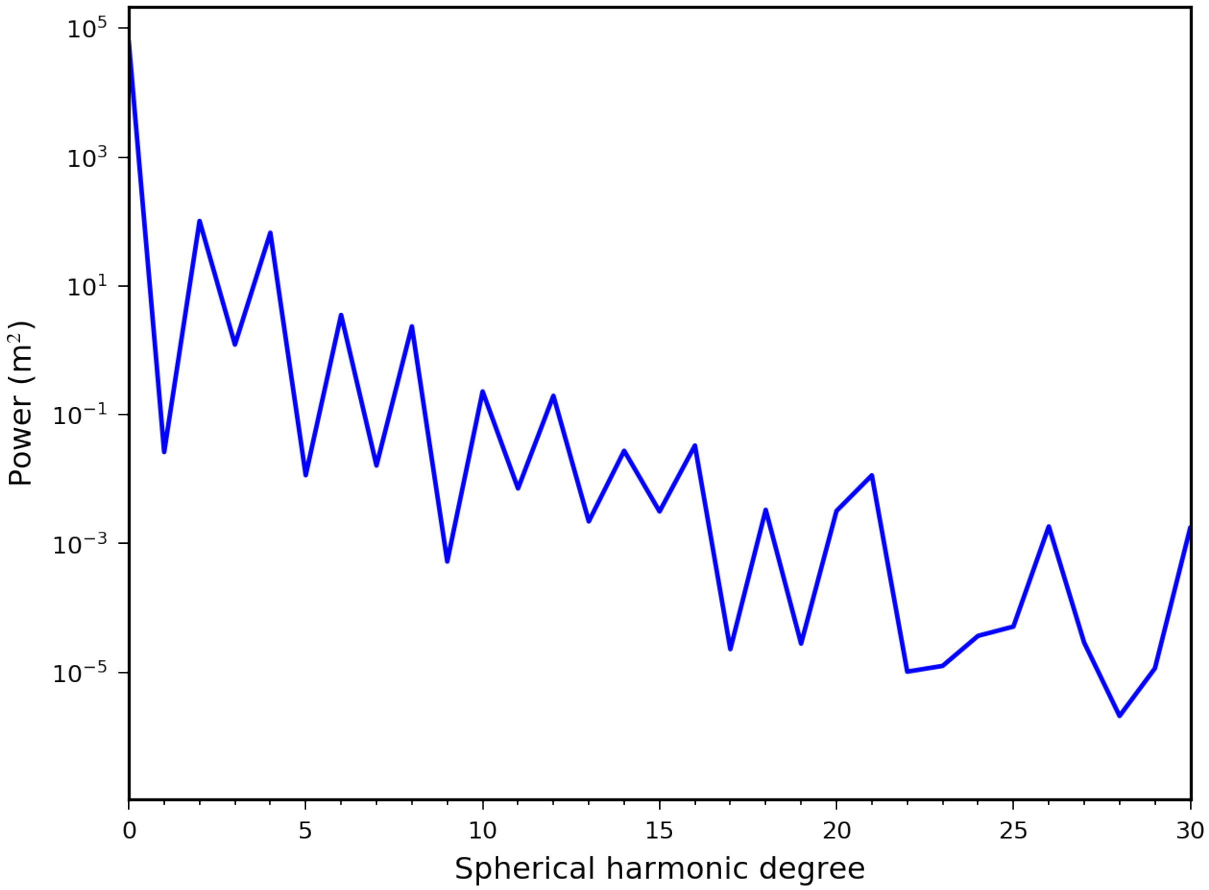

Fig. S6. The global zonal spherical harmonic coefficients. The high degree-2 term highlights the three regions of the model: northern hemisphere, southern hemisphere, and equatorial region.

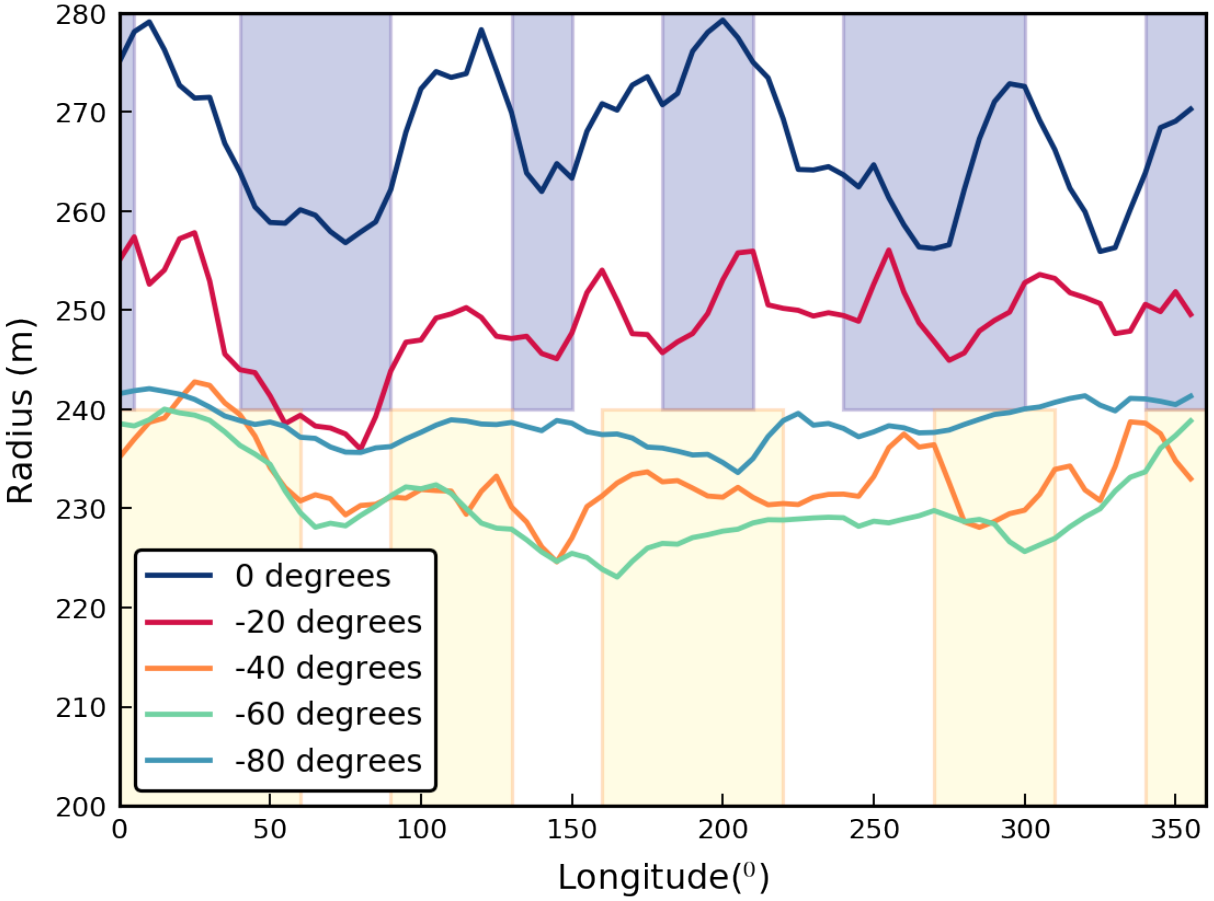

Fig. S7. The relationship between north-south ridges and low-albedo regions in the southern hemisphere. Blue shading shows the approximate locations of southern low-albedo regions as mapped in Fig. 7, and yellow shading indicates the locations of the ridges as identified in (*8*). Profiles of southern radii along latitudinal lines are overlaid.

Table S1. The largest boulders on Bennu. Geographic coordinates and dimensions for all boulders ≥ 30 m on Bennu as measured from the OLA DTM.

Latitude (◦)	Longitude (◦)	Long Dimensions (m)	Cross Dimension (m)	Geometric Height (m)	Volume Equivalent Spherical Diameter (m)
-63.5	146.1	33.8	31.0	7.9±0.9	25.5±0.2
-61.2	347.4	32.2	20.2	8±0.6	23.6±0.4
-59.0	287.9	29.5	19.3	7.7±1	23.4±0.03
-47.0	39.4	27.9	20.8	6.1±0.4	19.3±0.04
-44.7	126.2	47.7	39.8	27.4±0.7	53.2±0.2
-42.8	273.4	41.1	31.6	13.7±0.7	33.3±0.5
-40.3	261.2	30.4	19.6	7.8±0.5	15.9±0.4
-31.3	43.1	51.6	29.5	6±0.6	26.1±0.7
-29.6	48.3	29.9	10.1	4.4±0.4	11.9±0.3
-25.9	25.2	92.3	72.9	19.1±2.3	65.6±0.5
-25.8	3.2	40.5	34.1	16.8±1.6	37.1±0.2
-19.0	254.9	47.5	29.1	10.6±2.3	29.5±0.2
-16.1	300.2	29.3	9.4	5±0.6	11.6±11.6
-8.5	192.0	21.7	17.4	4.7±0.4	14.9±0.1
18.6	18.5	32.7	28.7	16.4±1	39.5±0.2
43.4	128.1	41.6	13.6	9.5±1	19.9±0.5
-83.7	203.2	38.5	33.7	8.2±0.9	28.7±0.1

Movie S1. A rendered rotation movie of the OLA GDTM of Bennu.

Movie S2. DTM of the boulder shown in Fig. 6B. False colors represent height difference from the best fit plane to each DTM and are provided as visual aid.

Movie S3. DTM of the boulder shown in Fig. 6C. False colors represent height difference from the best fit plane to each DTM and are provided as visual aid.

Movie S4. DTM of the boulder shown in Fig. 6D. False colors represent height difference from the best fit plane to each DTM and are provided as visual aid.

Movie S5. DTM of the boulder shown in Fig. 6D. False colors represent height difference from the best fit plane to each DTM and are provided as visual aid.
